# Comprehensive Analysis and Prediction of HER2-Targeted Therapy Insensitivity Among HER2-Positive Breast Cancer Patients Undergoing Neoadjuvant Treatment

**DOI:** 10.3390/cancers18060989

**Published:** 2026-03-18

**Authors:** Qingyao Shang, Zian Lin, Jennifer Plichta, Samantha Thomas, Meishuo Ouyang, Sheng Luo, Xin Wang

**Affiliations:** 1 Department of Breast Surgical Oncology, National Cancer Center/National Clinical Research Center for Cancer/Cancer Hospital, Chinese Academy of Medical Sciences and Peking Union Medical College, Beijing 100021, China; 2 Department of Biostatistics & Bioinformatics, Duke University School of Medicine, 2424 Erwin Road, Suite 1102, 11082 Hock Plaza, Durham, NC 27705, USA; 3 Department of Biostatistics, School of Public Health, University of Michigan, Ann Arbor, MI 48109, USA; 4 Duke Cancer Institute, Duke University School of Medicine, Durham, NC 27710, USA; 5 Department of Surgery, Duke University School of Medicine, Durham, NC 27705, USA

**Keywords:** HER2+ breast cancer, neoadjuvant treatment, targeted therapy, prediction model

## Abstract

Purpose: HER2-targeted therapy has been incorporated into the standard neoadjuvant treatment (NAT) regimen for HER2-positive early-stage breast cancer, yet a subset of patients have shown a limited pathological response. This study aimed to evaluate clinicopathological factors associated with NAT sensitivity and to develop a predictive model. Methods: This retrospective study included 13,004 HER2-positive breast cancer patients from the National Cancer Database (2010–2022) who received neoadjuvant chemotherapy plus HER2-targeted therapy. Pathological complete response (pCR) was defined as no residual invasive carcinoma in the breast and axillary lymph nodes (ypT0/is, ypN0). NAT sensitivity was additionally defined using clinical-to-pathologic stage migration according to the AJCC 8th edition criteria. Baseline characteristics and overall survival (OS) were compared between NAT-sensitive and NAT-insensitive groups. A multivariable logistic regression model was developed based on age, clinical T stage, clinical N stage, histologic subtype, tumor grade, and hormone receptor (HR) status. Model performance was assessed using the area under the receiver operating characteristic curve and calibration curves. Results: Among the patients included, 3660 (28.1%) achieved pCR. Based on the predefined stage-based criteria, 10,451 (80.4%) were classified as NAT-sensitive and 2553 (19.6%) as NAT-insensitive. NAT-insensitive patients were older and more likely to present with clinical T1c and node-negative disease, whereas NAT-sensitive patients more frequently had higher clinical T and N stages. HR-positive and lower tumor grades were significantly associated with treatment insensitivity. NAT-insensitive patients demonstrated significantly worse OS compared with NAT-sensitive patients (*p* < 0.001). The predictive model showed acceptable discrimination with AUCs of 0.762 in the training cohort and 0.776 in the validation cohort, demonstrating good calibration. Conclusions: NAT sensitivity in HER2-positive early-stage breast cancer exhibited substantial biological and clinical heterogeneity in real-world practice. A younger age, higher clinical stage, invasive ductal histology, higher tumor grade, and HR-negative status were associated with improved responses. A predictive model based on routinely available baseline variables demonstrated reasonable performance for estimating treatment sensitivity, supporting its potential utility for baseline risk stratification pending external validation.

## Simple Summary

Neoadjuvant therapy combining chemotherapy with HER2-targeted drugs has become a standard treatment for many patients with HER2-positive early-stage breast cancer. However, a proportion of patients have shown limited response to this treatment and may subsequently experience less favorable long-term outcomes. Identifying patients who are less likely to benefit before treatment begins could help clinicians optimize treatment strategies. In this study, we analyzed data from 13,004 patients with HER2-positive breast cancer who received neoadjuvant therapy using the National Cancer Database, a large nationwide oncology registry in the United States. We examined clinical and pathological factors associated with treatment responses and exploratorily constructed a prediction model using routinely available baseline information. Our findings have highlighted substantial variability in treatment sensitivity and could provide a practical tool that may help estimate the likelihood of treatment responses and facilitate baseline risk stratification.

## 1. Background

HER2-positive breast cancer accounts for approximately 15–20% of invasive breast cancers and is characterized by aggressive biological behavior [1]. The incorporation of anti-HER2-targeted therapy has dramatically improved outcomes in this subtype [2]. For patients with early-stage disease and high-risk features, neoadjuvant treatment (NAT) consisting of chemotherapy combined with dual-targeted therapy has become the standard of care [3,4]. According to the most recent National Comprehensive Cancer Network (NCCN) guidelines [5], neoadjuvant therapy (NAT) consisting of chemotherapy combined with HER2-targeted agents is recommended for patients with tumors ≥ cT2 or clinically node-positive disease, while it may be considered for selected patients with cT1c, cN0 HER2-positive disease, as it facilitates tumor downstaging, increases breast-conservation rates, and enables response-guided postoperative management [6,7].

Over the past two decades, NAT for HER2-positive early breast cancer has undergone a profound transformation. Around 2010, pivotal trials such as GeparQuattro and NOAH established the addition of trastuzumab to chemotherapy as the backbone of NAT, significantly increasing pathological complete response (pCR) rates while reducing recurrence and mortality [8,9]. These findings marked the beginning of an era for neoadjuvant-targeted therapy in early-stage disease [10]. However, despite substantial improvements, approximately 15–25% of patients treated with trastuzumab-based regimens still experienced disease relapse, likely reflecting an incomplete blockade of HER family signaling pathways [11,12]. Building on the survival advantage observed with dual HER2 blockade in the metastatic setting of the CLEOPATRA trial [13], subsequent neoadjuvant studies evaluated the addition of a second HER2-targeted agent to trastuzumab and chemotherapy. Trials incorporating pertuzumab or lapatinib demonstrated significantly higher pCR rates compared with single-agent HER2 blockade (95% CI 0.56–0.84; *p* < 0.001), thereby establishing dual HER2 inhibition as the standard of care for high-risk HER2-positive disease [4,14]. The KATHERINE trial demonstrated that patients with residual invasive disease after NAT derived substantial benefit from postoperative escalation to the antibody-drug conjugate trastuzumab emtansine (T-DM1), reinforcing the principle of tailoring adjuvant therapy according to pathological response [15]. Building on this concept, the development of next-generation antibody–drug conjugates, such as trastuzumab deruxtecan (T-DXd), has further expanded the therapeutic options and continues to reshape the treatment landscape [16]. Collectively, these advances have established a dynamic, biology-driven, response-adapted treatment paradigm in HER2-positive early-stage breast cancer.

However, despite these therapeutic advances, a substantial proportion of patients fail to achieve meaningful tumor regression following neoadjuvant HER2-targeted therapy [17]. These treatment-insensitive patients often experience inferior long-term outcomes and may not derive optimal benefit from standard neoadjuvant strategies.

In this study, we analyzed data from a large-scale, nationally representative cancer database to characterize clinicopathological features associated with response to neoadjuvant HER2-targeted therapy. We aimed to develop and validate a multivariable model using routinely available baseline variables to estimate the likelihood of treatment sensitivity, thereby facilitating early risk stratification and supporting individualized treatment decision-making in clinical practice.

## 2. Methods

### 2.1. Data Source and Study Design

This real-world retrospective study was based on data from the National Cancer Database (NCDB) [18]. We identified female patients diagnosed with early-stage HER2-positive invasive breast cancer between 2010 and 2022 who received neoadjuvant systemic therapy consisting of chemotherapy combined with HER2-targeted therapy.

Eligible patients were required to have complete information on demographic characteristics, clinical staging, histological type, hormone receptor (HR) status, tumor grade, treatment response to NAT, and documented survival outcomes. Patients with missing data in any of the predefined variables were excluded from the final analysis.

### 2.2. Definition of Neoadjuvant Sensitivity

NAT response was conventionally assessed using pCR, residual cancer burden (RCB), or Miller–Payne (MP) grading systems, which provided standardized measures of tumor regression following therapy [19,20,21,22]. Given the variables accessible in the present dataset, pCR was selected as an indicator of NAT sensitivity and was defined as ypT0/is and ypN0.

In addition, NAT sensitivity was also defined based on changes in clinical tumor (cT) and nodal (cN) stages according to the AJCC 8th edition staging system, consistent with prior studies utilizing clinical-to-pathologic stage migration as a surrogate indicator of neoadjuvant response [22,23,24]. Patients were considered NAT-sensitive if either the pathologic T stage (ypT) or the pathologic N stage (ypN) decreased compared with the corresponding baseline clinical stage (cT or cN), without evidence of stage progression at the other site.

### 2.3. Statistical Analysis

Descriptive statistics were used to summarize the baseline characteristics. Continuous variables were reported as means with standard deviations and compared using Student’s *t*-test. Categorical variables were presented as counts with percentages and compared using the chi-square test.

Baseline variables were selected for multivariable logistic regression model based on both the clinical relevance and observed differences between NAT-sensitive and NAT-insensitive groups. Key clinicopathological factors known to be associated with treatment response, including age, clinical T stage, clinical N stage, histologic subtype, tumor grade, and hormone receptor (HR) status, were considered in the model’s construction. Due to the potential biological association between tumor grade and HR status, their interaction term was explored and included in the logistic regression model. The likelihood ratio test was conducted to evaluate the statistical significance of this interaction. The predictive model was developed by randomly splitting the study population into a training set (70%) and internal validation set (30%). The internal validation set was a hold-out set. Model discrimination was assessed using the area under the receiver operating characteristic curve (AUC). Calibration curves were also drawn in both the training dataset and the internal validation dataset to determine whether the model produced well-calibrated probabilities.

A Kaplan–Meier (KM) curve was drawn to compare overall survival (OS) between NAT-sensitive and NAT-insensitive patients. The OS was compared using the log-rank test. A multivariable cox proportional hazards model was also fitted to adjust for confounders and quantify the prognostic value of NAT insensitivity more robustly. All statistical analyses were performed using R software (version 4.2.2), and a two-sided *p* value < 0.05 was considered statistically significant.

## 3. Results

A total of 13,004 female patients with early-stage HER2-positive breast cancer who received neoadjuvant HER2-targeted therapy were included in this analysis. A total of 3660 patients (28.1%) achieved pCR following NAT (Table 1). Patients who achieved pCR were younger than those without pCR (mean age: 52.64 vs. 55.07 years). Baseline clinical stage distributions were generally comparable between groups. cT2 tumors were the most common category in both groups (57.0% in the pCR group vs. 58.3% in the non-pCR group). Similarly, patients in the pCR group demonstrated a modestly higher prevalence of advanced nodal disease (cN2-3: 8.9% vs. 6.6%), whereas cN0 disease was slightly more frequent among non-pCR patients (62.1% vs. 58.9%).

The histologic subtype differed between groups. Invasive ductal carcinoma (IDC) was more common in the pCR group (91.1% vs. 87.5%), whereas invasive lobular carcinoma (ILC) and other non-ductal histologies were more frequently observed in the non-pCR group. The tumor grade also showed clear differences. Poorly differentiated tumors (grade 3) were substantially more prevalent among patients achieving pCR (65.0% vs. 55.3%), while grade 1 and grade 2 tumors were more common in the non-pCR group.

In addition, HR status differed markedly between groups. An HR-negative status was more frequently observed among patients who achieved pCR than among those who did not(43.8% vs. 26.2%), whereas an HR-positive status was predominant among non-pCR patients (73.8% vs. 56.2%). No substantial differences were observed in the primary tumor sites or laterality between the two groups.

Based on the predefined criteria for treatment sensitivity, 10,451 patients (80.4%) were classified as NAT-sensitive, whereas 2553 patients (19.6%) were classified as NAT-insensitive. Significant differences in the baseline characteristics were observed between the NAT-insensitive and NAT-sensitive groups (Table 2).

NAT-insensitive patients were older (mean age: 56.4 vs. 53.9 years, *p* < 0.001). In terms of race distribution, white patients were more frequently observed in the NAT-insensitive group (80.3% vs. 77.9%), whereas Asian patients were more common in the NAT-sensitive group (6.0% vs. 4.2%) (*p* = 0.002). Ethnicity also differed significantly between groups, with a higher proportion of non-Hispanic patients in the NAT-insensitive group (91.0% vs. 89.4%, *p* = 0.020).

Marked differences were noted in baseline clinical stage. Patients in the NAT-insensitive group were substantially more likely to present with clinical T1c tumors (52.4% vs. 14.2%), while NAT-sensitive patients more frequently had more advanced T stage disease, including T2 (62.3% vs. 40.2%), T3 (16.0% vs. 4.6%), and T4 tumors (7.6% vs. 2.7%) (*p* < 0.001). Among T1c insensitive patients, the nodal status was predominantly cN0 (n = 1088), followed by cN1 (n = 239), and with only a small proportion presenting as cN2 (n = 11) or cN3 (n = 1). In terms of tumor differentiation, 92 cases were grade 1, 702 were grade 2, and 545 were grade 3. A total of 1120 patients were observed to be HR-positive, while 219 were HR-negative. Similarly, the clinical N stage also differed significantly. NAT-insensitive patients more commonly had cN0 disease (73.5% vs. 58.2%), whereas higher nodal stages (cN1-3) were more prevalent in the NAT-sensitive group (*p* < 0.001).

The histologic subtype, tumor grade, and hormone receptor status differed significantly between the two groups (all *p* < 0.001). NAT-sensitive patients were more likely to have IDC and poorly differentiated (grade 3) tumors, whereas NAT-insensitive patients presented more frequently with non-ductal histologies and grade 1/2 tumors. In addition, an HR-positive status was substantially more common in the NAT-insensitive group. No significant differences were observed in the primary tumor sites (*p* = 0.400) or laterality (*p* = 0.457).

**Figure 1:**
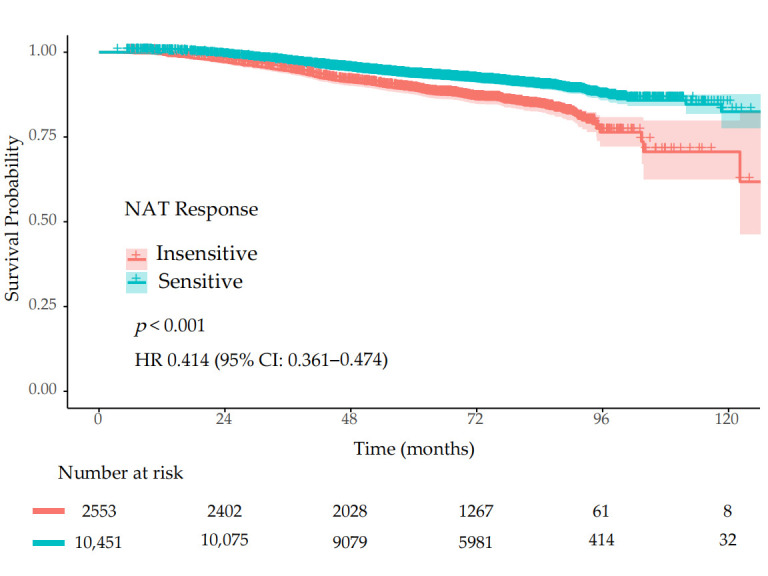
Kaplan–Meier curves comparing overall survival between NAT-sensitive and NAT-insensitive patients.

Kaplan–Meier analysis demonstrated significantly worse OS among NAT-insensitive patients compared with NAT-sensitive patients (Figure 1, *p* < 0.001). After adjustment for confounders, NAT sensitivity remained independently associated with improved survival (HR 0.414, 95% CI 0.361–0.474).

Based on both clinical relevance and observed differences between NAT-sensitive and NAT-insensitive groups, variables including age, clinical T and N stages, histologic subtype, tumor grade, HR status, race, ethnicity, and interactions between tumor grade and HR status were selected for inclusion in the multivariable logistic regression model to predict NAT sensitivity and adjust for confounders. The model parameters and ORs are presented in Table 3.

**Figure 2:**
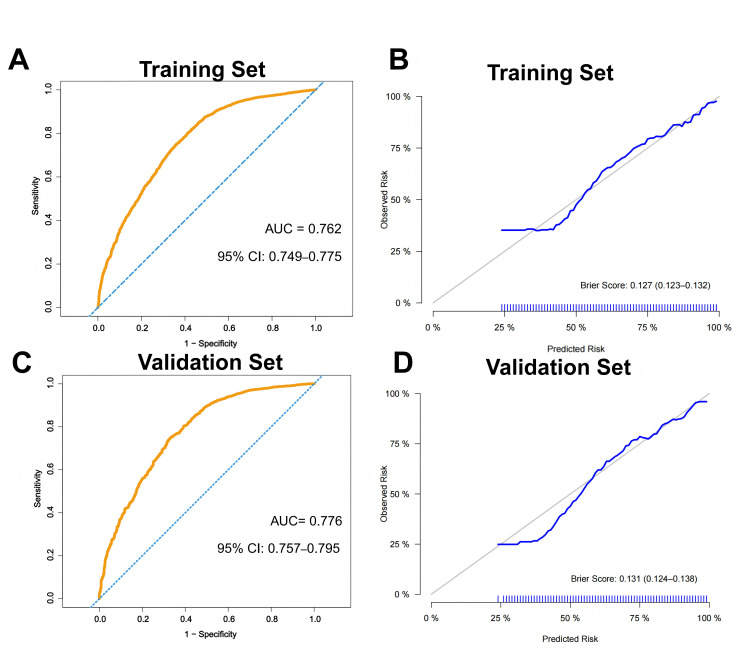
Discrimination and calibration performance of the predictive model. (**A**) Receiver operating characteristic (ROC) curve for the training cohort. (**B**) Calibration curve in the training cohort. (**C**) ROC curve for the internal validation cohort. (**D**) Calibration curve in the internal validation cohort.

The model demonstrated good discriminatory ability in both the training (AUC = 0.762, 95% CI 0.749–0.775, Figure 2A) and internal validation sets (AUC = 0.776, 95% CI 0.757–0.795, Figure 2C). Calibration curves demonstrated good agreement between predicted and observed probabilities in both cohorts, supporting its robustness and internal generalizability (Figure 2B,D). The Brier score was 0.127 (95% CI 0.123–0.132) in the training set and 0.131 (95% CI 0.124–0.138) in the validation set.

## 4. Discussion

For patients with HER2-positive early-stage breast cancer who have been selected to receive NAT, a combination of chemotherapy and HER2-targeted agents has been the current standard of care [25]. However, a subset of patients have failed to respond adequately to treatment and subsequently experience inferior long-term outcomes, deriving limited benefit from standard neoadjuvant strategies.

Pathological response to NAT has traditionally been evaluated using several standardized criteria, including pCR, MP grading, and RCB [19,20]. Among these, pCR has remained the most widely adopted endpoint in clinical trials due to its established association with improved event-free and overall survival [3,4]. In previous randomized clinical trials (RCT), pCR rates in HER2-positive early-stage breast cancer have varied substantially, approximately ranging from 30% to 65%, depending on the treatment intensity and HER2-targeted strategy. Earlier studies incorporating single-agent trastuzumab plus chemotherapy demonstrated relatively modest pCR rates, such as 31.7% in GeparQuattro and 42% in the NOAH trial [8,9]. With the introduction of a dual HER2 blockade, response rates improved significantly. In the NeoALTTO trial, dual inhibition with trastuzumab and lapatinib achieved a pCR rate of 51.3%, compared with 29.5% in the trastuzumab-alone arm [26]. Similarly, the NeoSphere study reported a pCR rate of 45.8% with pertuzumab, trastuzumab, and docetaxel [4]. More recently, the KRISTINE trial demonstrated a pCR rate of 55.7% with standard chemotherapy combined with trastuzumab and pertuzumab, which was superior to the 44.4% observed with T-DM1 plus pertuzumab [27]. Our real-world cohort showed a pCR rate of 28.1% (n = 3660). Compared with the above RCTs, the notably lower pCR rate likely reflected multiple factors, including treatment heterogeneity, potential underutilization of a dual HER2 blockade during earlier years of the study period, variability in staging accuracy, and differences between RCT populations and routine clinical practice. Our study period spanned 2010–2022, during which neoadjuvant standards evolved substantially, transitioning from single-agent trastuzumab-based regimens to routine dual HER2 blockades. In addition, registry-based datasets such as the NCDB do not provide granular information regarding specific anti-HER2 regimens, chemotherapy intensity, treatment duration, or adherence, all of which may influence response rates.

Consistent with the prior literature, we observed that patients achieving pCR were generally younger and more likely to have HR-negative, high-grade, and invasive ductal tumors [28,29]. These clinicopathological characteristics have been well-established predictors of increased responsiveness to HER2-targeted NAT and can reflect underlying tumor biology, including higher proliferative activity and greater HER2 pathway dependence.

However, pCR represents a binary endpoint and cannot fully capture intermediate or partial responses. To better characterize the broader spectrum of treatment sensitivity, we further defined NAT-sensitive and NAT-insensitive groups based on reductions in both primary tumor and nodal stages. Using this surrogate staging-based definition, we identified distinct clinicopathological features associated with treatment sensitivity, including younger ages, higher clinical stages, invasive ductal histologies, higher tumor grades, and an HR-negative status.

Notably, in our study, the majority of patients classified as NAT-insensitive presented with clinical T1c tumors. According to the most recent NCCN guidelines [5], NAT consisting of chemotherapy combined with HER2-targeted agents has been recommended for patients with tumors ≥ cT2 or clinically node-positive disease, while it may be considered for selected patients with cT1c/cN0 HER2-positive disease. However, given the relatively lower likelihood of treatment sensitivity observed in this subgroup and the potential delay of definitive surgery associated with NAT, whether patients with cT1c disease should routinely receive NAT should remain open to further discussion. Over the past decade, treatment strategies for T1c HER2-positive breast cancer have increasingly focused on therapy de-escalation rather than intensification. The APT trial demonstrated excellent long-term outcomes with adjuvant paclitaxel plus trastuzumab in patients with small, node-negative HER2-positive tumors, supporting fewer intensive approaches in selected patients [30]. Similarly, the ATEMPT trial evaluated adjuvant T-DM1 as a de-escalated strategy in stage I HER2-positive disease, further reinforcing that many T1c tumors may achieve favorable outcomes without aggressive systemic therapy [31]. Against this background, the use of NAT in T1c tumors within real-world practice could likely reflect heterogeneity in risk assessment and institutional preference rather than uniform biological aggressiveness. Importantly, a substantial proportion exhibited intermediate- to high-grade histology, and a large subset were HR-positive. Given that HR-positive/HER2-positive tumors have been known to achieve lower pCR rates compared with HR-negative disease, intrinsic biological subtype differences likely contributed to the observed variability in response patterns.

On the basis of these findings, we constructed an exploratory predictive model using routinely available pre-treatment clinical and core needle biopsy pathological variables to estimate the likelihood of NAT sensitivity. The multivariable logistic regression model demonstrated acceptable discrimination, with AUCs of 0.762 and 0.776 in the training and internal validation cohorts, respectively, and calibration curves also showed good fitting of the model. This model was intended as a tool for baseline risk stratification rather than a substitute for clinical decision-making.

Several limitations should be acknowledged. First, the retrospective design and reliance on registry data preclude causal inference and may introduce misclassification or unmeasured confounding. Second, because response rates vary substantially across regimens, the NCDB does not capture detailed information regarding specific HER2-targeted regimens (e.g., trastuzumab alone versus dual HER2 blockade), chemotherapy intensity, treatment adherence, or post-neoadjuvant escalation strategies, which may substantially influence pathological response. Third, although we incorporated pCR analysis, other validated measures of response, such as residual cancer burden or Miller–Payne grading, were unavailable. Fourth, staging-based definitions of sensitivity may be influenced by variability in baseline clinical staging accuracy. Finally, our predictive model underwent only internal validation and should therefore be regarded as exploratory rather than ready for routine clinical application. In addition, the possibility of model overfitting cannot be completely excluded, and further external validation in independent cohorts is required.

This study has several notable strengths. By analyzing a large-scale, real-world cohort, we were able to describe patterns of NAT response in HER2-positive early-stage breast cancer within routine clinical practice and show their clear association with long-term survival. The inclusion of both tumor- and nodal-stage changes allowed for a clinically grounded assessment of treatment heterogeneity. In addition, we constructed a predictive model using routinely available pre-treatment variables, which may help inform baseline risk assessments and provide a foundation for future validation studies.

## 5. Conclusions

This study identified key clinicopathological factors associated with response to neoadjuvant HER2-targeted therapy in HER2-positive early-stage breast cancer. A younger age, negative hormone receptor status, higher tumor grade, and IDC histology were associated with improved treatment sensitivity and higher pCR rates. A predictive model based on routinely available pre-treatment variables showed acceptable performance for estimating the likelihood of treatment sensitivity, although further external validation is required. These findings have highlighted the heterogeneity of neoadjuvant response in routine practice and underscored the importance of continued efforts to refine risk stratification strategies in this population.

## Figures and Tables

**Table 1:** Baseline clinicopathological characteristics of patients stratified by pathological complete response to neoadjuvant treatment.

Variable	Non-pCR Patients	pCR Patients
*n*	9344 (71.9%)	3660 (28.1%)
Age (mean ± SD)	55.07 ± 12.68	52.64 ± 11.61
Race		
White	7357 (78.7%)	2832 (77.4%)
Black	1279 (13.7%)	484 (13.2%)
Asian	505 (5.4%)	235 (6.4%)
Others	203 (2.2%)	109 (3.0%)
Ethnicity		
Non-Hispanic	8395 (89.8%)	3272 (89.4%)
Hispanic/Other	949 (10.2%)	388 (10.6%)
Clinical T stage		
1c	2025 (21.7%)	793 (21.7%)
2	5447 (58.3%)	2086 (57.0%)
3	1257 (13.5%)	532 (14.5%)
4	615 (6.6%)	249 (6.9%)
Clinical N stage		
0	5799 (62.1%)	2157 (58.9%)
1	2926 (31.3%)	1177 (32.2%)
2	367 (3.9%)	184 (5.0%)
3	252 (2.7%)	142 (3.9%)
Histology		
IDC	8173 (87.5%)	3334 (91.1%)
ILC	321 (3.4%)	63 (1.7%)
IPC	266 (2.8%)	77 (2.1%)
Others	584 (6.3%)	186 (5.1%)
Grade		
1	388 (4.2%)	66 (1.8%)
2	3790 (40.6%)	1215 (33.2%)
3	5166 (55.3%)	2379 (65.0%)
Primary site		
Central	504 (5.4%)	188 (5.1%)
Non-Central	6366 (68.1%)	2500 (68.3%)
Overlapping	2474 (26.5%)	972 (26.6%)
Laterality		
Right	4628 (49.5%)	1807 (49.4%)
Left	4716 (50.5%)	1853 (50.6%)
Hormone Receptor status		
Negative	2452 (26.2%)	1604 (43.8%)
Positive	6892 (73.8%)	2056 (56.2%)

IDC, Invasive Ductal Carcinoma; ILC, Invasive Lobular Carcinoma; IPC, Invasive Papillary Carcinoma.

**Table 2:** Comparison of characteristics of patients with HER2+ early-stage breast cancer who are insensitive and sensitive to neoadjuvant treatment.

Variable	Sensitive Group	Insensitive Group	*p*-Value
*n*	10,451 (80.4%)	2553 (19.6%)	
Age (mean ± SD)	53.9 ± 12.3	56.4 ± 12.7	<0.001
Race			0.002
White	8140 (77.9%)	2049 (80.3%)	
Black	1419 (13.6%)	344 (13.5%)	
Asian	632 (6.0%)	108 (4.2%)	
Others	260 (2.5%)	52 (2.0%)	
Ethnicity			0.020
Non-Hispanic	9344 (89.4%)	2323(91.0%)	
Hispanic/Other	1107 (10.6%)	230(9.0%)	
Clinical T stage			<0.001
1c	1479 (14.2%)	1339 (52.4%)	
2	6506 (62.3%)	1027 (40.2%)	
3	1671 (16.0%)	118 (4.6%)	
4	795 (7.6%)	69 (2.7%)	
Clinical N stage			<0.001
0	6080 (58.2%)	1876 (73.5%)	
1	3485 (33.3%)	618 (24.2%)	
2	500 (4.8%)	51 (2.0%)	
3	386 (3.7%)	8 (0.3%)	
Histology			<0.001
IDC	9347 (89.4%)	2160 (84.6%)	
ILC	275 (2.6%)	109 (4.3%)	
IPC	252 (2.4%)	91 (3.6%)	
Others	577 (5.5%)	193 (7.6%)	
Grade			<0.001
1	309 (3.0%)	145 (5.7%)	
2	3805 (36.4%)	1200 (47.0%)	
3	6337 (60.6%)	1208 (47.3%)	
Primary site			0.400
Central	557 (5.3%)	135 (5.3%)	
Non-Central	7098 (67.9%)	1768 (69.3%)	
Overlapping	2796 (26.8%)	650 (25.5%)	
Laterality			0.457
Right	5189 (49.7%)	1246 (48.8%)	
Left	5262 (50.3%)	1307 (51.2%)	
Hormone Receptor status			<0.001
Negative	3578 (34.2%)	478 (18.7%)	
Positive	6873 (65.8%)	2075 (81.3%)	

IDC, Invasive Ductal Carcinoma; ILC, Invasive Lobular Carcinoma; IPC; Invasive Papillary Carcinoma.

**Table 3:** Logistic regression with interaction identifying independent predictors of neoadjuvant treatment sensitivity.

Variable	Estimate (β)	OR (95%CI)	*p*-Value
Intercept	1.22	3.37 (1.23, 9.23)	0.018 *
Age (year)	−0.02	0.98 (0.98, 0.99)	<0.001 **
Race (Ref: White)			0.204
Black	−0.12	0.89 (0.75, 1.05)	0.168
Asian	0.21	1.23 (0.93, 1.62)	0.141
Other	0.02	1.02 (0.68, 1.53)	0.928
Ethnicity: (Ref: Non-Hispanic)			
Hispanic/Other	−0.02	0.99 (0.81, 1.20)	0.881
Clinical T stage (Ref: T1c)			<0.001 **
T2	1.68	5.37 (4.75, 6.07)	<0.001 **
T3	2.32	10.19 (7.97, 13.03)	<0.001 **
T4	2.26	9.56 (6.84, 13.36)	<0.001 **
Clinical N stage (Ref: N0)			<0.001 **
N1	0.28	1.32 (1.16, 1.51)	<0.001 **
N2	0.57	1.77 (1.21, 2.57)	0.003
N3	1.68	5.34 (2.58, 11.06)	<0.001 **
Histology (Ref: IDC)			<0.001 **
ILC	−0.29	0.75 (0.56, 1.02)	0.067
IPC	−0.40	0.67 (0.48, 0.93)	0.017
Others	−0.48	0.62 (0.49, 0.78)	<0.001 **
Grade (Ref: 1)			
2	0.54	1.72 (0.64, 4.64)	0.282
3	0.32	1.38 (0.52, 3.63)	0.517
HR status: (Ref: Negative)			
Positive	−0.81	0.45 (0.17 1.21)	0.113
Grade * HR status			<0.001 **
2 * Positive	−0.36	0.70 (0.25, 1.97)	0.501
3 * Positive	0.27	1.31 (0.48, 3.62)	0.597

IDC: Invasive Ductal Carcinoma; ILC: Invasive Lobular Carcinoma; IPC: Invasive Papillary Carcinoma. *: *p* < 0.05; **: *p* < 0.001. β indicates the direction and magnitude of association with NAT sensitivity, where β > 0 indicates higher likelihood of sensitivity.

## Data Availability

The dataset analyzed in this study was obtained from the National Cancer Database (NCDB). All analytical results generated during this study are presented in the manuscript.

## Abbreviations

HER2	Human Epidermal Growth Factor Receptor 2
NAT	Neoadjuvant Treatment
pCR	Pathological Complete Response
OS	Overall Survival
AUC	Area Under the Curve
NCDB	National Cancer Database
HR	Hormone Receptor
ypT	Pathologic Tumor Stage (post-treatment)
ypN	Pathologic Node Stage (post-treatment)
cT	Clinical Tumor Stage (pre-treatment)
CI	Confidence Interval
KM	Kaplan–Meier
ROC	Receiver Operating Characteristic
IDC	Invasive Ductal Carcinoma
ILC	Invasive Lobular Carcinoma
IPC	Invasive Papillary Carcinoma
